# Traumatic hemorrhage and chain of survival

**DOI:** 10.1186/s13049-023-01088-8

**Published:** 2023-05-24

**Authors:** Rana K. Latif, Sean P. Clifford, Jeffery A. Baker, Rainer Lenhardt, Mohammad Z. Haq, Jiapeng Huang, Ian Farah, Jerrad R. Businger

**Affiliations:** 1 Department of Anesthesiology and Perioperative Medicine, University of Louisville School of Medicine, University of Louisville Hospital, 530 S. Jackson St., Louisville, KY 40202 USA; 2 Paris Simulation Center, Office of Medical Education, University of Louisville School of Medicine, Louisville, KY USA; 3 Outcomes Research Consortium, Cleveland, OH USA; 4 Department of Emergency Medicine, University of Louisville School of Medicine, Louisville, KY USA; 5 Department of Cardiovascular & Thoracic Surgery, Cardiovascular Innovation Institute, University of Louisville, Louisville, KY USA; 6 The Center for Integrative Environmental Health Sciences, University of Louisville, Louisville, KY USA; 7 Department of Pharmacology and Toxicology, University of Louisville School of Medicine, Louisville, KY USA; 8 Division of Infectious Diseases, Department of Medicine, Center of Excellence for Research in Infectious Diseases (CERID), University of Louisville, Louisville, KY USA

**Keywords:** Traumatic hemorrhage, Diagnostic imaging in trauma, Damage control resuscitation, Damage control surgery, Chain of survival algorithm in trauma

## Abstract

Trauma is the number one cause of death among Americans between the ages of 1 and 46 years, costing more than $670 billion a year. Following death related to central nervous system injury, hemorrhage accounts for the majority of remaining traumatic fatalities. Among those with severe trauma that reach the hospital alive, many may survive if the hemorrhage and traumatic injuries are diagnosed and adequately treated in a timely fashion. This article aims to review the recent advances in pathophysiology management following a traumatic hemorrhage as well as the role of diagnostic imaging in identifying the source of hemorrhage. The principles of damage control resuscitation and damage control surgery are also discussed. The chain of survival for severe hemorrhage begins with primary prevention; however, once trauma has occurred, prehospital interventions and hospital care with early injury recognition, resuscitation, definitive hemostasis, and achieving endpoints of resuscitation become paramount. An algorithm is proposed for achieving these goals in a timely fashion as the median time from onset of hemorrhagic shock and death is 2 h.

**Supplementary Information:**

The online version contains supplementary material available at 10.1186/s13049-023-01088-8.

## Introduction

According to the National Trauma Institute, trauma is the number one cause of death among Americans between the ages of 1 and 46 years, costing $670 billion a year in health care dollars [1]. Hemorrhage is estimated to account for more than 60,000 deaths in the United States and 1.5 million deaths worldwide each year resulting in nearly 75 million years of life lost [2]. The median time from onset of hemorrhagic shock to death is 2 h [3]. Following central nervous system injury, hemorrhage is the leading cause of death in trauma patients [4–6]; however, hemorrhage is amenable to interventions for reducing morbidity and mortality [7, 8]. Among those with severe multisystem trauma, early in-hospital mortality is increased by continued hemorrhage, which leads to a vicious triad of coagulopathy, hypothermia, and acidosis in the setting of incomplete or inappropriate resuscitation [9–11].

The military experience including research from the wars in Iraq and Afghanistan affirmed the need for improved methods of hemorrhage control [12, 13]. Department of Defense analysis of battlefield mortality demonstrated that one in four pre-hospital combat deaths and one in two in-hospital combat deaths were potentially preventable [14, 15]. This article aims to review recent advances in our understanding of the pathophysiology of traumatic hemorrhage, the role of diagnostic imaging modalities in the timely identification of hemorrhage sources, the principles of damage control resuscitation (DCR), as well as definitive hemostasis and damage control surgery (DCS). Finally, the endpoints of trauma resuscitation are summarized, and a chain of survival algorithm is proposed to achieve these endpoints in a timely manner. Although our understanding of the pathophysiology and management principles related to traumatic hemorrhage continues to improve, many questions remain unanswered in improving survival, and further study is needed.

## Pathophysiology

**Fig. 1:**
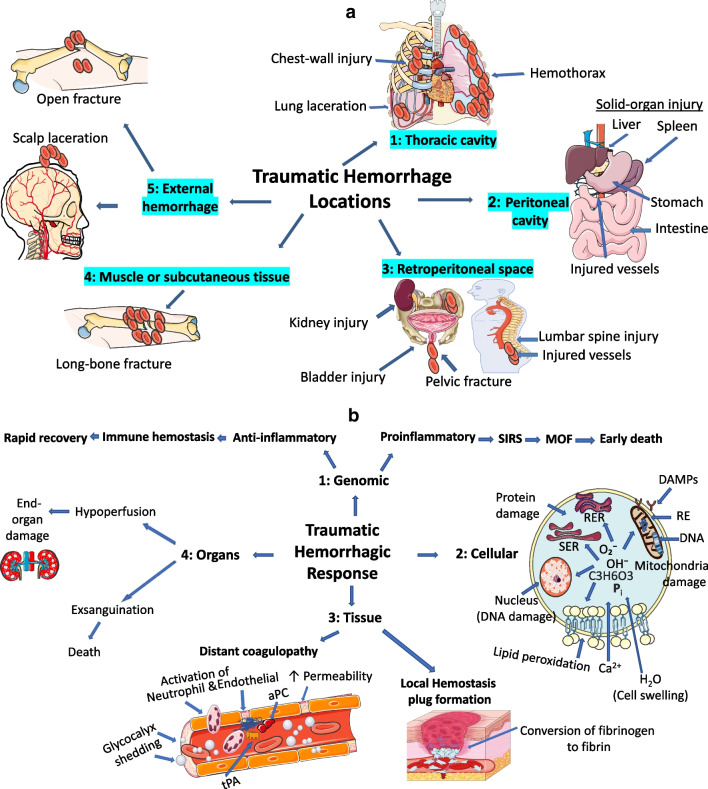
Pathophysiology of traumatic hemorrhagic shock. **a** Traumatic hemorrhage five locations. **b** Traumatic hemorrhagic response. (1) Genomic response. Up-regulated anti-inflammatory genes with rapid recovery. Up-regulated pro- inflammatory genes leads to complications and death. (2) Cellular response. Anerobic metabolism with damage to mitochondria, smooth endoplasmic reticulum (SER) and rough endoplasmic reticulum (RER), leading to cellular homeostasis failure. (3) Tissue response. Local hemostatic plug formation with conversion of fibrinogen to fibrin. Distant coagulopathy with hyperfibrinolysis and diffuse coagulopathy. (4) Organ response. Moderate hemorrhage with end-organ damage and Exsanguination leading to death. *aPC* activated protein C, *CARS* compensatory anti-inflammatory response syndrome, *DAMPs* damage-associated molecular patterns, *DNA* deoxyribonucleic acid, *P*_*i*_ inorganic phosphate, *C*_*3*_*H*_*6*_*O*_*3*_ lactic acid, *MOF* multi organ failure, O_2_^–^, OH^–^, oxygen radicals, *RE* respiratory enzymes, *SIRS* systemic inflammatory response syndrome, *tPA* tissue plasminogen activator, *↑* increased

In terms of large-volume bleeding, the following body locations or surface sources must be considered: thoracic cavity, peritoneal cavity, retroperitoneal space (e.g., pelvic fracture), muscle or subcutaneous tissue (e.g., long-bone fracture) and external hemorrhage (e.g., scalp laceration, open fracture site) (Fig. 1a) [16]. Hemorrhage and hemorrhagic shock cause inadequate oxygen delivery and activate several homeostatic mechanisms designed to preserve perfusion to vital organs. These complex events occur at the genomic, cellular, tissue, and whole-organ levels (Fig. 1b).

At the genomic level, proinflammatory and anti-inflammatory innate immunity genes are up-regulated while adaptive immunity genes are simultaneously down-regulated in the early hours post-injury [17]. In addition, it has been demonstrated that the systemic inflammatory response syndrome (SIRS) and compensatory anti-inflammatory response syndrome (CARS) occur simultaneously rather than sequentially, as previously thought [17, 18]. In patients that survive, it is likely that anti-inflammatory innate immunity genomic changes translate into the phenotypic changes of innate SIRS, which is followed by relative immunosuppression, termed compensatory anti-inflammatory response syndrome (CARS), and eventual recovery without complications [17]. In patients with poor outcomes, further investigation is required regarding whether proinflammatory changes in gene expression reflect an ongoing or repeated inflammatory stimulus leading to multi-organ failure (MOF) [18]. Current understanding is most consistent with a non-resolving inflammation hypothesis among these patients which leads to SIRS, MOF, and early death [19].

At the cellular level, hemorrhage results in inadequate oxygen delivery, and as cells transition to anaerobic metabolism, there is accumulation of oxygen radicals (O_2_^–^, OH^–^), inorganic phosphate (P_i_) and lactic acid (C_3_H_6_O_3_). This causes lipid peroxidation of membranes with increased permeability to Ca^2+^ and subsequent breakdown of mitochondria, smooth endoplasmic reticulum (SER) and rough endoplasmic reticulum (RER) [20, 21]. Cellular disruption causes release of damage-associated molecular patterns (known as DAMPs or alarmins), including mitochondrial DNA and formyl peptides, and incites a systemic inflammatory response similar to sepsis [22, 23]. Anaerobic respiration ultimately leads to mitochondrial dysfunction resulting in diminished ATP supplies, cellular homeostasis failure, and eventual cell death through necrosis from membrane rupture, apoptosis, or necroptosis [24].

At the tissue level, hemorrhage, and shock cause both adaptive and maladaptive changes within the vascular endothelium and blood. At the hemorrhagic site, the endothelium and blood act synergistically; the clotting cascade and platelets are activated leading to formation of a hemostatic plug [25, 26]. However, remote from the site of bleeding, sympathoadrenal activation and the mounting oxygen debt induces endotheliopathy with shedding of the glycocalyx barrier leading to excess autoheparinization with activation of protein C (aPC) and inactivation of factors V and VIII to prevent microvascular thrombosis [26–28]. Similarly, release of tissue plasminogen activator (tPA) results in increased plasmin activity leading to pathologic hyperfibrinolysis and diffuse coagulopathy [26, 28]. Hemorrhage induced decreases in platelet number and function as well as margination also contribute to the coagulopathy [29–31].

At the whole-organ level, moderate hemorrhagic shock causes vasoconstriction with hypoperfusion leading to end-organ damage in survivors. However, severe shock with exsanguination can cause cerebral anoxia and fatal arrhythmias leading to death [3, 32]. Iatrogenic factors like overzealous resuscitation with cold, acidic crystalloid not only dilute the concentration of clotting factors but also exacerbate the ‘lethal triad’ of heat loss, acidosis, and coagulopathy (Fig. 1) [33–36].

## Diagnostic imaging in traumatic hemorrhage

**Fig. 2:**
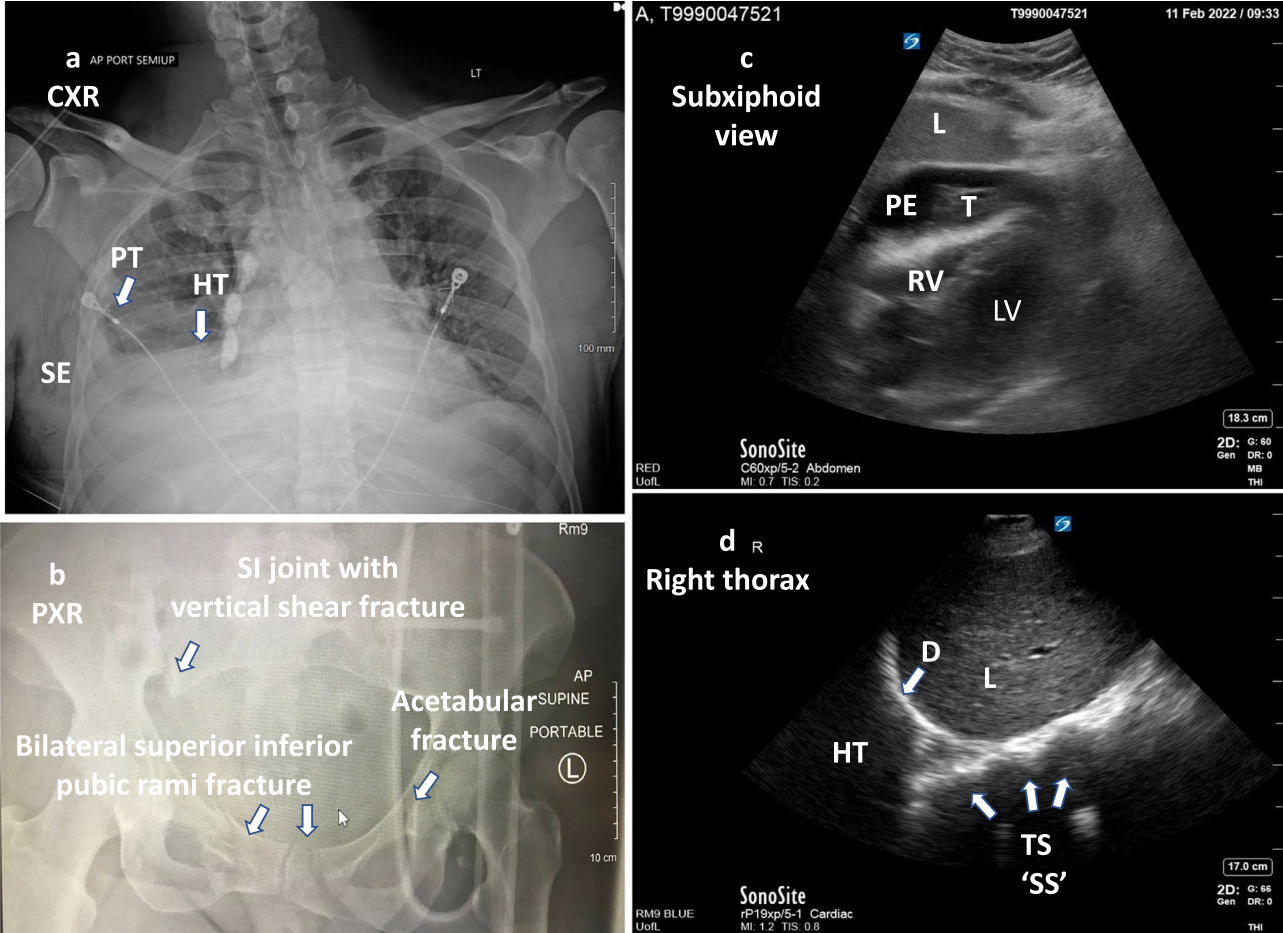
**a** Chest X-ray AP view. Right sided hemothorax, right lateral pneumothorax and subcutaneous emphysema. **b** Pelvic AP view. Widening of right sacroiliac joint with right sacral fracture and vertical shift (potential vascular injury), bilateral superior inferior pubic rami fracture (risk for bladder injury), left acetabular fracture. **c** Subxiphoid view of the heart (2D). Large pericardial effusion causing tamponade. **d** Right thoracic view at the diaphragm with a right hemothorax. Thoracic spine visualized above the diaphragm (spine sign). Normally, the thoracic spine is obscured by air within the lung. *D* diaphragm, *HT* hemothorax, *L* liver, *LV* left ventricle, *PE* pericardial effusion, *PT* pneumothorax, *RV* right ventricle, *SE* subcutaneous emphysema, *SI* sacroiliac joint, *SS* spine sign, *T* thrombus, *TS* thoracic spine

**Fig. 3:**
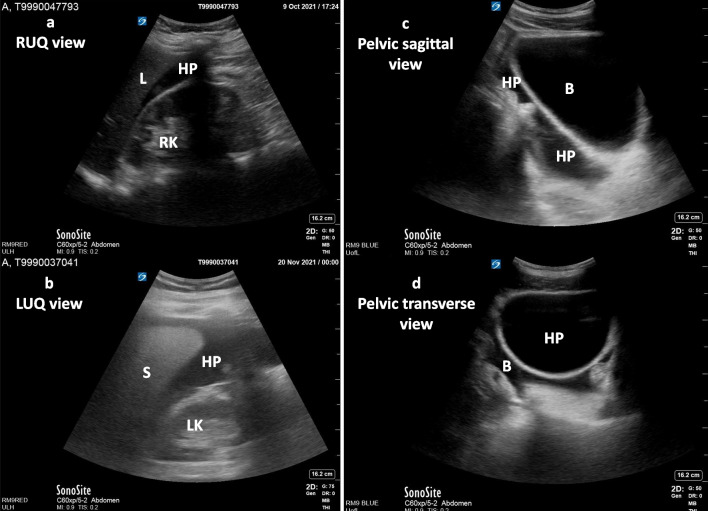
**a** Right upper quadrant view (RUQV) of the abdomen. Anechoic hemoperitoneum in the hepatorenal space. **b** Left upper quadrant view (LUQV) of the abdomen. Anechoic hemoperitoneum in the splenorenal space. **c** Pelvic sagittal view. Anechoic hemoperitoneum cephalad and posterior to the bladder. **d** Pelvic transverse view. Anechoic hemoperitoneum posterior to the bladder. *B* bladder, *HP* hemoperitoneum, *L* liver, *LK* left kidney, *RL* right kidney, *S* spleen

Imaging plays a vital role in the identification of hemorrhagic sources as well as the response to therapeutic interventions. Diagnostic imaging techniques of critical importance in identifying specific pathology (modality no 1 to 7) and assessing cardiovascular hemodynamics (modality no 8 to 11) for patients presenting with traumatic hemorrhage are summarized in Table 1 along with pertinent findings and key values. Portable chest, pelvic radiographs (Fig. 2a, b) and the Focused Assessment with Sonography for Trauma (FAST) are the standard of care in the initial bedside evaluation of traumatic injuries [16]. As compared to X-rays and FAST, computed tomography (CT) are more sensitive in evaluating important anatomical details and altered hemodynamics [37–39]. In hemodynamically stable patients with multiple injuries, CT technology can comprehensively detect trauma to the chest, abdomen, and pelvis, as well as active bleeding with sensitivity and specificity approaching 100% [38, 40–43]. Multidetector CT scan is considered the gold standard in the assessment of cardiac, vascular, small bowel and mesenteric injuries [40, 42, 43]. In trauma patients, the FAST utilizes a standard order of views or windows to evaluate the pericardial, peritoneal, and pleural cavities [44]. FAST can determine the presence of pathologic hemopericardium or pericardial effusion (sensitivity 83.3% to 100% and specificity 94% to 99.7%) (Fig. 2c, Additional file 1: Video 1), hemothorax (sensitivity 83% to 92% and specificity 98% to 100%) (Fig. 2d, Additional file 2: Video 2), intraabdominal hemoperitoneum (sensitivity 63 to 100 percent) (Fig. 3a, b; Additional file 3: Video 3, Additional file 4: Video 4), and pelvic hemorrhage (Fig. 3c, d; Additional file 5: Video 5, Additional file 6: Video 6) [44–51].

**Table 1:** Diagnostic imaging in traumatic hemorrhage

Parameter assessed in trauma	Imaging modalities	Required views	Pertinent findings and key values
(1) Hemothorax (Pleural cavity) ATLS [16]	Chest radiograph (Fig. 2a)	AP (Upright preferred)	Blunting of costophrenic angle or partial or complete opacification of the affected half of the thorax
(2) Pelvic hemorrhage (a) AP compression fracture (15–20%) (b) Lateral compression fracture (60–70%) (c) Vertical shear fracture (5–15%) (d) Combined fracture mechanism Cullinane et al. [127]	Pelvic radiograph (Fig. 2b)	AP	(a) Pubic diastasis, disrupted pelvic ring (b) Internal rotation with injury risk to bladder and urethra (c) Vertical displacement of sacroiliac joint (d) Combined
(3) Multisystem trauma Fang et al. [40], Cinquantini et al. [41]	CT/ MDCT	2D images of a “slice” of the body. Can be used to construct 3D images	Comprehensively detect trauma to the chest, abdominal, pelvic, and active bleeding
(4) Hemopericardium (Pericardial tamponade) Klein et al*.* [46]	FAST (2D) M-mode Doppler (Fig. 2c, Additional file 1: Video 1)	Subcostal/subxiphoid, parasternal long axis (PSLA), parasternal short axis (PSSA) and apical four chamber (A4C)	Tamponade criteria: Large fluid quantification, > 1 cm RA systolic collapse > 30% of the cardiac cycle RV diastolic collapse
(5) Hemothorax (Pleural cavity) Brooks et al*.* [50]	FAST (2D) (Fig. 2d, Additional file 2: Video 2)	RUQV: lower right thorax LUQV: lower left thorax (Angle the probe up above the diaphragm into chest cavity)	Anechoic area between the diaphragm and the parietal pleura within the costophrenic recess
(6) Intraperitoneal free fluid (Abdomen) Holmes et al*.* [51]	FAST (2D) (Fig. 3a, Additional file 3: Video 3) (Fig. 3b, Additional file 4: Video 4)	RUQV (Hepatorenal view) LUQV (Perisplenic view)	Anechoic area (free fluid) between the liver and right kidney (Morisons’s pouch) Anechoic area surrounding the spleen and obscuring the interface between the spleen and left kidney
(7) Intraperitoneal free fluid (Pelvic) Cullinane et al*.* [127]	FAST (2D) (Fig. 3c, Additional file 5: Video 5) (Fig. 3d, Additional file 6: Video 6)	Sagittal view Transverse view	Aided by fluid-filled bladder Anechoic area in the rectouterine space or pouch of Douglas (female) or rectovesical space (male)
(8) Intravascular volume status: IVC size/collapsibility, for RAP Rudski et al*.* [54], Brennan et al. [55]	2D (Fig. 4a, b) (Additional file 7: Video 7)	Visualization throughout the respiratory cycle	Size ≤ 2.1 cm; collapses > 50% during sniff = RAP 0–5 mm Hg Size > 2.1 cm; collapses > 50% during sniff = RAP 5–10 mm Hg Size > 2.1; collapses < 50% during sniff = 10- RAP 20 mm Hg
(9) Intravascular volume status and cardiac function: LV and RV chamber size, areas, and volumes Lang et al. [56]	2D Volume (Fig. 4c, d) (Additional file 8: Video 8) Function (Additional file 9: Video 9)	Parasternal long axis (PSLA), parasternal short axis (PSSA) and apical four chamber (A4C	Normal ranges: LVIDD 3.9–5.9 cm LVEDV 46–150 mL LVESV 14–61 mL LVEF > 51% RV FAC ≥ 35%
(10) Cardiac stroke volume & function (LV): LVOT VTI Ristow et al. [58]	2D; pulsed Doppler	Apical 5 chamber or 3 chamber views Optimal Doppler alignment Pulse wave Doppler at LVOT	Normal value: VTI ≥ 18 cm
(11) RV function: TAPSE RV Tissue Doppler S’ Rudski et al. [54]	M-mode (TAPSE) Tissue Doppler (RV S ‘)	Optimal Apical four chamber view, alignment with TV annulus, M mode for TAPSE, Tissue Doppler for S’	Normal value: TAPSE ≥ 16 mm RV S ‘ ≥ 10 cm/sec

Modality # 1 to 7 are diagnostic, modality # 8 to 11 are for volume status and cardiac functions

*AP* anteroposterior, *CT* computed tomography, *2D* two-dimensional, *3D* three-dimensional, *FAST* Focused Assessment with Sonography for Trauma, *IVC* inferior vena cava, *LUQV* left upper quadrant view, *LV* left ventricular, *LVEDV* LV end diastolic volume, *LVEF* LV ejection fraction, *LVESV* LV end-systolic volume, *LVIDD* LV internal diameter at end-diastole, *LVOT* LV outflow tract, *MDCT* multidetector computed tomography, *RAP* right atrial pressure, *RUQV* right upper quadrant view, *RV* right ventricular, *RV FAC* right ventricular fractional area change, *RV S'* RV systolic excursion velocity, *TAPSE* tricuspid annular plane systolic excursion, *TV* tricuspid valve, *VTI* velocity time integral

**Fig. 4:**
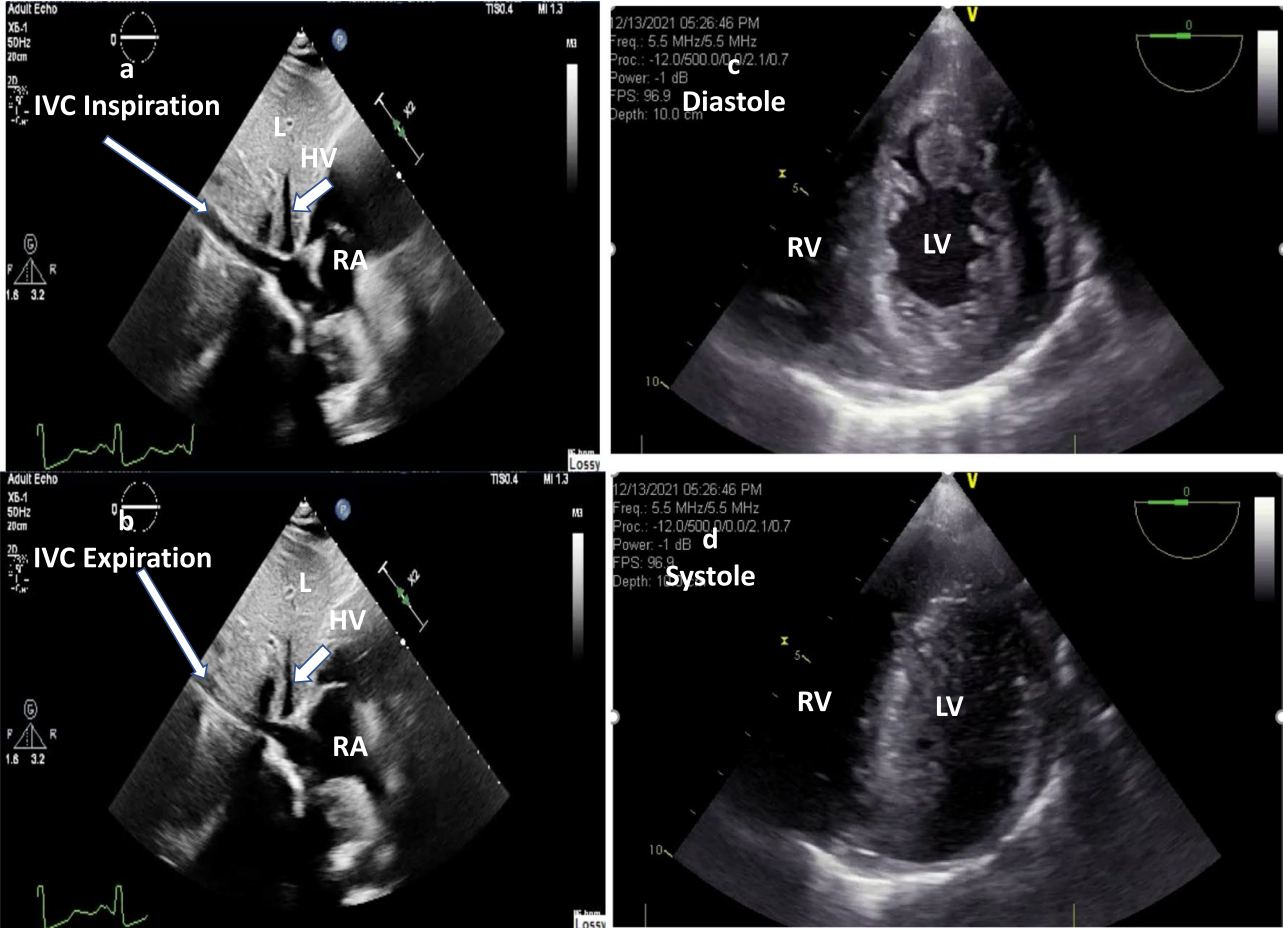
**a** TTE sagittal view of IVC long axis during inspiration; **b** IVC during expiration. Collapses > 50% with respiration provide insight into the fluid status of an adult trauma patient. **c** TEE transgastric short axis view during diastole; **d** systole. Severe left ventricular hypovolemia and papillary muscle kissing sign during systole. *HP* hepatic vein, *IVC* inferior vena cava, *L* liver, *LV* left ventricle, *RA* right atrium, *RV* right ventricle

Hypovolemia can be identified using measurements of both the inferior vena cava (IVC) diameter and IVC collapsibility index (IVCCI) with respiration (Fig. 4a, b; Additional file 7: Video 7) [52–55]. Among these hypovolemic patients, the echocardiogram may demonstrate a small and under-filled left ventricle with preserved or hyperdynamic function (Fig. 4c, d; Additional file 8: Video 8) [56]. In the peri-operative as well as postoperative period, the cause of shock and required treatment with fluid and/or inotropes therapy can be monitored using left ventricle internal diameter at end-diastole (LVIDD), LV end-diastolic volume (LVEDV), left ventricle end-systolic volume (LVESV), left ventricle ejection fraction (LVEF), and right ventricle fraction area change (RVFAC) (Table 1, modality no 8 to 11 and Additional file 9: Video 9) [54, 56–58].

## Chain of survival

**Fig. 5:**
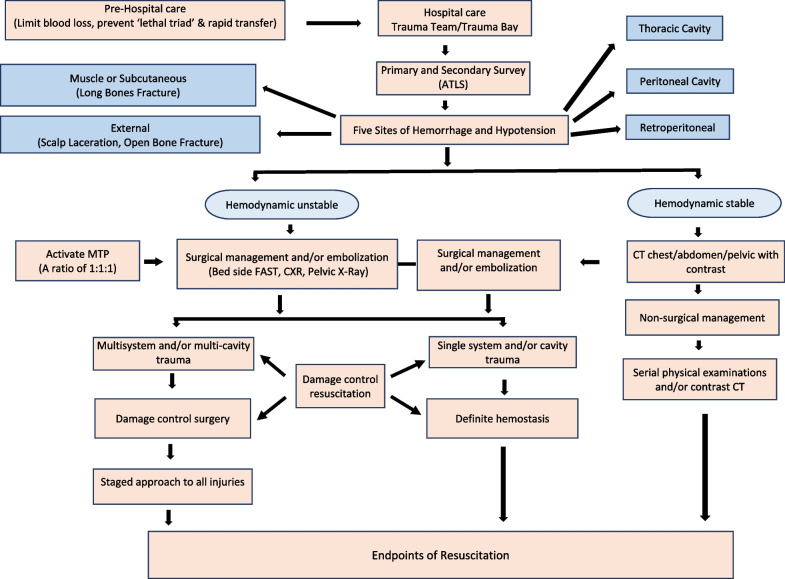
Traumatic hemorrhage and chain of survival. ATLS, Advanced Trauma Life Support; CXR, chest X-ray; CT, computed tomography; FAST, Focused Assessment with Sonography in Trauma; MTP, Massive Transfusion Protocol; 1:1:1, equal amounts of packed red cells, fresh frozen plasma, and platelets

The chain of survival for patients with severe hemorrhage begins with goals of primary prevention. Following a traumatic event, focus shifts to prehospital hemorrhage control; once the patient arrives at the hospital, timely recognition of shock, resuscitation, definitive hemostasis, and achieving endpoints of resuscitation factor into the outcome. Primary prevention involves developing programs for industry and community-based violence prevention as well as increasing workplace and motor vehicle safety awareness and compliance with safety gear (seatbelt and helmet). The community-based trauma education includes B-Con (Bleeding Control) Basic or ‘Stop the Bleeding’ course, (a course aimed at educating first responders and the general public on how to stop severe bleeding and potentially save lives in an emergency) [59], Prehospital Trauma Life Support (PHTLS) Course [60], Rural Trauma Team Development Course (RTTDC) [61], and Advanced Trauma Life Support (ATLS) Student Course [16]. The prehospital and hospital chain of survival care involves applying the principles of the Damage Control Resuscitation (DCR), definite hemostasis and Damage Control Surgery (DCS), and achieving endpoints of hemostasis in a timely fashion (Fig. 5).

### Damage control resuscitation (DCR)

- **Pre-hospital care**

Priorities for prehospital care include (1) minimizing further blood loss, (2) providing limited/delayed fluid resuscitation with permissive hypotension, (3) preventing hypothermia, and (4) rapidly transporting the patient to a facility that can provide definitive care. Tourniquet application proximal to the sites of hemorrhage in the extremities, pelvic binder placement for suspected pelvic fracture, and hemostatic dressing therapy for bleeding wounds at junctional locations (e.g., groin, axilla) can minimize blood loss and save lives [62–67]. A study by Bickell et al. examining the effects of delaying resuscitation (i.e., withholding intravenous fluid until the moment of definitive hemostasis) demonstrated improved survival, fewer complications, and reduced length of hospital stay compared with immediate resuscitation in patients with penetrating torso injuries [68]. Maintaining the principle of ‘permissive hypotension’ (systolic blood pressure 80–90 mm Hg) with low volume crystalloid boluses offers early survival advantages in blunt urban trauma patients [69].

The presence of concomitant moderate to severe traumatic brain injury (TBI) may complicate the management. Hypotension (systolic blood pressure < 90 mmHg) and hypoxia (PaO_2_ < 60 mmHg) were associated with a higher likelihood of a poor outcome in TBI patients [70, 71]. The Brain Trauma Foundation (BTF) and World Society of Emergency Surgery (WSES) guidelines for the management of severe TBI recommend maintaining SBP at ≥ 100 [72, 73]. However, hypotensive patients with TBI frequently have other traumatic injuries to internal organs, lungs, limbs, or the spinal cord [16], and DCR (temporarily maintaining SBP < 90 mm Hg, prevent clot disruption and re-bleeding) with immediate intervention to control severe hemorrhage may be lifesaving in these patients [68, 69].

Trauma in geriatric patients may create a special diagnostic challenge as their myocardium is less sensitive to catecholamines, and they have increased systemic vascular resistance. This results in a less profound tachycardic and hypotensive response to hemorrhage which could lead to clinical misinterpretation [74]. Among geriatric trauma patients, heart rates above 90 beats per minute and systolic blood pressure less than 110 mmHg correlate with increased mortality [74]. Older individuals are more likely to have chronic heart and lung disease. Many geriatric trauma patients may be receiving anticoagulants, antiplatelet agents, beta blockers, calcium channel blockers, and glucocorticoids for heart and lung disease. Pre-injury anticoagulants, beta blockade and glucocorticoid therapy has been shown to increase the odds of death among these trauma patients [75–77]. The United States Centers for Disease Control (CDC) suggests direct transport to a trauma center for any injured patient 65 or older with a systolic blood pressure < 110 mmHg [78].

Prehospital blood product resuscitation is undergoing investigation in military trauma patients with promising results [79]. In civilian trauma, the RePHILL trial was inconclusive [80] and the two large, randomized control trials (PAMPER and COMBAT) examining the benefit of prehospital plasma transfusion were contradictory. The PAMPER trial favored prehospital transfusion [81] and the COMBAT showed no benefit [82] but the post hoc analysis from 2 randomized clinical trials support a survival benefit in plasma transfusion group when transport times are longer than 20 min [83]. The CRASH-2 trial favors early administration of TXA (within 3 h of injury) to reduce the risk of death in bleeding trauma patients and is highly cost-effective [84].

The past decade has seen tremendous development of local/topical and injectable hemostatic agents for prehospital and hospital hemorrhage control [85]. Several intracavitary foams are under development to stop bleeding from a noncompressible abdominal hemorrhage [85]. ResQFoam is currently under clinical trial review to demonstrate the safety, effectiveness, and benefit-risk profile for the treatment of emergent, exsanguinating, intra-abdominal hemorrhage resulting in Class III or IV hemorrhagic shock due to trauma (https://clinicaltrials.gov/ct2/show/NCT02880163). ClotFoam, another type of intracavitary foam, is under a phase 1 clinical trial for hemostasis in liver bleeding (https://clinicaltrials.gov/ct2/show/NCT02264730). Several new hemostatic agents are under development, each with their own pros and cons based on the type of injury, severity of bleeding, wound size and configuration, location on the body, accessibility to the bleeding site, and the patient’s coagulation function [85].

Prevention of the lethal triad of hypothermia, acidosis, and coagulopathy is of paramount importance. Hypothermia should be managed using warmed blankets and warmed intravenous fluids; [86] however, these prehospital measures should not delay the transfer of a patient from the scene to the hospital [87]. In military combat, the morbidity and mortality were lower when the critically injured were transported in ≤ 60 min [87]. During prolong field care (more than 4 h), hemorrhage control (tourniquets) with blood transfusion, airway, and ventilatory support are potentially lifesaving and highly time-critical, resource-intensive interventions [88].

b. **Hospital care**

In-hospital management starts with assembling a highly functional, multidisciplinary trauma team with individuals drawn from the specialties of anesthesia, emergency medicine, surgery, nursing, and radiology with support from the blood bank, laboratory, and patient care assistants. An experienced physician team leader receives input from the individual providers and coordinates assessment and management [89]. The Advanced Trauma Life Support (ATLS) student manual emphasizes early recognition of hemorrhagic shock, rapidly controlling the source of hemorrhage, and restoring the patient’s intravascular volume and oxygen-carrying capacity. Trauma patients can lose up to 30 percent of their blood volume before significant drops in blood pressure are manifested (Additional file 10) [16]. On arrival in the emergency department, the initial assessment and treatment are performed simultaneously in the seriously injured patient according to standard ATLS protocols. The primary survey begins with stabilization of the patient's **a**irway, **b**reathing, **c**irculation,** d**isability assessment and **e**xposure/**e**nvironmental control (ABCDEs; i.e., primary survey) [16, 90]. During the secondary survey, patients are evaluated from head-to-toe based upon their hemodynamic status and the mechanism of injury (blunt and/or penetrating) with consideration that hypotension is secondary to hemorrhage unless proven otherwise [16].

An initial aggressive resuscitation approach should be pursued with all adult trauma patients with hemorrhage, including geriatric trauma patients [91]. Vascular access is obtained as rapidly as possible with two large-bore (16-gauge or larger) intravenous (IV) lines. When difficulty arises in placing IV lines, ultrasound guided peripheral venous catheterization or central line catheterization (size 8 French), intraosseous devices, and distal saphenous vein cutdowns offer effective alternatives [92–94]. Initial laboratory evaluation includes blood for cross match, complete blood count (baseline hemoglobin or hematocrit and platelets), complete metabolic panel, coagulation studies [thromboelastography (TEG) and rotational thromboelastometry (ROTEM) when available], serum lactate, and arterial blood gas (serum bicarbonate for base deficit) [30, 95].

Subsequent emergency care includes activation of massive-transfusion (MT) protocols and transfusing equal amounts of packed red cells, fresh frozen plasma, and platelets in a ratio of 1:1:1 (PROPPR trial) during the early empiric phase of resuscitation. Pharmaceutical adjuncts such as calcium and tranexamic acid are important components in optimizing hemostasis [84, 96–101]. The Eastern Association for the Surgery of Trauma (EAST) guidelines compare MT with a *high ratio* (1:1:1) of fresh frozen plasma, platelets, and red blood cells (relatively more plasma and platelet) vs. a *low ratio* 1:1:2 (relatively less plasma and platelets) [101]. The EAST qualitative analysis indicated an early mortality benefit to targeting a *high ratio* [97, 102] due to a more frequent achievement of hemostasis [97], decreased death from truncal hemorrhage [103] or exsanguination [97]. Before the 1970s, whole blood (WB) was the resuscitation fluid of choice for bleeding trauma patients [104]. The recent data from the military and civilian trauma literature suggesting better global hemostasis using WB rather than blood components has led to a renewed interest in WB [96, 101, 105]. The EAST guidelines recommend that patients in hemorrhagic shock would benefit from *high ratio* DCR, if not whole blood [101] while transitioning to a laboratory-based resuscitation strategy as results become available [97, 101].

During the early hours of hospital care, the MT contents or blood products contain the anticoagulant citrate, which the liver rapidly metabolizes in healthy persons. In patients receiving MT, citrate may become toxic with life-threatening hypocalcemia and progressive coagulopathy [100, 106]. Empirical calcium dosing (e.g., 1 g of calcium chloride after administration of 4 units of RBC and/or FFP) [106] should be paired with frequent measurements of electrolyte levels to prevent hypo or hypercalcemia. [100] In the first 6 h, the isotonic crystalloid administration should be limited to 3 L to reduce the risk of respiratory failure, acute kidney injury, abdominal and extremity compartment syndromes, coagulopathy, and possibly mortality [107–109]. Off-label use of procoagulant hemostatic adjuncts including activated recombinant factor VII, tranexamic acid, prothrombin complex concentrate, and fibrinogen concentrate should be based on careful interpretation of the original studies and current guidelines [101]. The presence of massive transfusion protocols along with their timely activation and damage-control resuscitation (DCR) provide a decrease in mortality in trauma patients [101, 110].

### Definitive hemostasis and damage control surgery (DCS)

Trauma patients with severe bleeding require timely, definitive hemostasis with surgery or angiography with embolization as prolonged time to hemostasis has been linked to increased blood-transfusion requirements and increased mortality [111, 112]. From the outset, it is important to identify the cavity with the most significant bleeding using diagnostic imaging or invasive modalities (FAST, DPL, thoracostomy) as poorly ordered surgical exploration delays definitive hemostasis and increases the risk of death [113]. Patients with extremity bleeding requiring a tourniquet and/or multicavity torso hemorrhage should not stay in the emergency department for more than 10 min [111].

Massive hemothorax (≥ 1500 ml) is identified during primary survey and initially treated with tube thoracostomy. Patient physiology should be the primary indications for surgical intervention rather than absolute numbers of initial or persistent output. Immediate bloody drainage of ≥ 20 mL/kg (approximately 1500 mL) or continuous bleeding of ≥ 3 mL/kg/hour (approximately 200 mL/hr for 2 to 4 h) may be considered an indication for surgical thoracotomy but adequate scientific evidence is lacking and need further research [16, 114, 115].

In abdominal trauma patients, surgical judgment and timing is important, and the following indications are commonly used to determine the need for laparotomy [116–118]: blunt abdominal trauma with hypotension and a positive FAST (Fig. 3a, b; Additional file 3: Video 3, Additional file 4: Video 4), or clinical evidence of intraperitoneal bleeding/peritonitis without another source of bleeding [16, 117–119]. Patients with penetrating wounds of the abdomen with associated hypotension, peritonitis, bleeding from the stomach (NG tube aspirate), rectum, genitourinary tract, or evisceration may require emergent laparotomy [120, 121]. Decisions on gunshot injuries are based on the trajectory, cavitation effect, and possible bullet fragmentation [120]. Gunshot wounds that by physical examination or routine imaging demonstrate penetration of the peritoneal cavity or viscera and vascular area of the retroperitoneum usually require laparotomy [120, 122]. Blunt or penetrating solid organ injury in hemodynamically stable patients can often be managed nonoperatively but should be admitted to the hospital for careful observation and evaluated by serial physical examinations and/or contrast-enhanced CT [122–126].

Patients with hypotension and pelvic fracture have high mortality rates and necessitate a team rescue effort of trauma surgeons, orthopedic surgeons, and interventional radiologists or vascular surgeons [127, 128]. The hemorrhage commonly involves disruption of the posterior osseous ligamentous complex (i.e., sacroiliac, sacrospinous, sacrotuberous, and fibromuscular pelvic floor), evidenced by a sacral fracture, a sacroiliac fracture, and/or dislocation of the sacroiliac joint [16, 127, 128]. Based on injury force, pelvic fractures are classified into four types (Table 1) and can predict those patients at high risk for massive hemorrhage [16, 127, 129]. Initial hemorrhage control is achieved through mechanical stabilization of the pelvic ring and external counter pressure by internal rotation of the lower limbs with application of a sheet or pelvic binder at the level of the greater trochanters of the femur [16]. Subsequent intervention can include pre-peritoneal packing, external fixator placement, and angiographic embolization can be used to control pelvic venous as well as arterial hemorrhage. [127, 128, 130]

The Resuscitative Endovascular Balloon Occlusion of the Aorta (REBOA) therapeutic intervention for hemorrhage is in the early stages of evaluation, but it may decrease the amount of bleeding distal to the occluded site and provide a window of opportunity for resuscitation and definitive hemorrhage control [131–134]. A consensus panel recommended utilization of REBOA in patients with an initial blood pressure of < 90 mmHg that do not respond or respond only transiently to resuscitation [135]. Controlled partial REBOA is now technically possible, and the use of partial REBOA is also supported by expert panel who suggest that the most viable tool in prolonging the potential use of REBOA while attempting to avoid the dangers of distal ischemia is early partial occlusion (transitioning to partial occlusion after a short period of full occlusion) [135]. REBOA is associated with significantly reduced mortality when compared with no method of aortic occlusion, but prolonged occlusion times are associated with increased mortality [133]. Further studies are needed to determine the tolerable duration of balloon inflation, type of the balloon, ideal timing of REBOA placement, and the eligible patients who may benefit from REBOA. Preclinical studies evaluating these and other REBOA techniques are ongoing [136].

Resuscitation and management of life-threatening injuries takes precedence over the extremity or musculoskeletal injury [16, 137]. It is essential to recognize and manage musculoskeletal arterial injuries, compartment syndrome, open fractures, crush injuries, and dislocations in a timely manner [16, 137]. The mortality rate for arterial injuries is 2.2% for upper extremity and 7.7% for lower extremity injuries [138]. A staged approach to hemorrhage control is utilized by applying direct pressure, splints, tourniquets (250 mm Hg in an upper extremity and 400 mm Hg in a lower extremity), and immediate operative surgical repair when musculoskeletal injury is the source of hemodynamic instability [137]. Hemorrhage from scalp wounds may be extensive and can be controlled by applying direct pressure, cauterization, and ligation of large vessels through appropriate usage of sutures, clips, or staples [139].

In the 1980s, the concept of ‘damage control surgery’ (DCS) was initiated in severely injured patients with multisystem trauma. This concept can be broken down into distinct phases [140, 141]. Phase 0 includes rapid transport and triage for treatment (eg, operating room, interventional suite). Phase 1 encompasses surgery to arrest the hemorrhage, limit contamination, and maintain optimal blood flow to vital organs and the extremities. Operative time is limited to minimize further exacerbation of the ‘lethal triad’ of coagulopathy, hypothermia, and acidosis. Phase 2 is resuscitation in the ICU, and Phase 3 is a staged approach to definite repair of all injuries based upon the patient's physiologic status (Additional file 10). Finally, Phase 4 involves closure of the abdomen or other soft tissue wounds and delayed complex reconstructive surgery, should the primary fascia closure not be achieved during the initial hospitalization [141]. DCR and DCS are associated with a survival advantage and shorter ICU length of stay in patients with severe hemorrhage [142]. The phases and principles of Damage Control Resuscitation (DCR) and Damage Control Surgery (DCS) as well as an algorithm to achieve these goals are summarized in Fig. 5.

### Endpoints of resuscitation

The traditional ATLS course standard of care markers of successful resuscitation includes restoration of normal blood pressure (BP), heart rate (HR), and urine output [16]. However, approximately 85% of severely injured trauma victims still have evidence of inadequate tissue oxygenation after normalization of BP, HR, and urine output based upon ongoing metabolic acidosis or gastric mucosal ischemia [143]. The Eastern Association for the Surgery of Trauma (EAST) guidelines for endpoints of resuscitation fall into 2 categories: global and regional [144]. The patients who achieve supranormal global oxygen delivery goals (cardiac index (CI) > 4.5 l/min/m^2^, oxygen delivery (DO2) > 600 ml/min/m^2^, and oxygen consumption (VO2) > 170 ml/min/m^2^) may have a better chance of survival than those who do not achieve these goals but there is no convincing evidence that attempting to attain these goals directly improves survival [145, 146]. Trying to achieve supranormal oxygen delivery goals can led to over-resuscitation, open abdomens, longer ventilation time and increased mortality [107, 108]. Other considerations for global endpoints of resuscitation include normalization of base deficit (normal range ± 2 mEq/L), lactic acid (normal range 0.5–1 mmol/L), and end-tidal carbon dioxide (normal range 35–45 mmHg) [11, 147], yet these values have failed to demonstrate conclusive survival advantage [144].

Regional endpoints include monitoring gastric ischemia using gastric tonometry or intramucosal pH (pHi, normal range ≥ 7.35) and sublingual pCO_2_ (normal range 45.2 ± 0.7 mm Hg). The normalization of pHi or pCO_2_ gap can predict better outcome [148–150]. Skeletal muscle and subcutaneous tissue pO_2_ (normal range 80–100 mmHg), pCO_2_ (normal range 35–45 mmHg), and pH (normal range 7.03 ± 0.02) can be monitored to demonstrate decreased regional blood flow by using near infrared spectroscopy or tissue electrodes [151, 152]. Preliminary data suggest that they may have potential for predicting risk of MOF and death after trauma [144]. Numerous resuscitation endpoint parameters have been studied but they failed to show clear mortality benefits and more work is needed [144].

## Discussion

Traumatic hemorrhagic represents a serious therapeutic problem that results in high patient mortality when not managed properly. The time from injury to hospital admission, diagnosis, resuscitation, and definite hemostasis should be as abbreviated as possible. The pathophysiology of traumatic hemorrhage is complex, and imaging modalities are important to identify the source of bleeding. The application of damage control resuscitation (DCR), definitive hemostasis, and damage control surgery (DCS) have shown promising results in trauma patients. The guidelines for endpoints of resuscitation are developed but too limited in their scope to show clear outcome benefit at this point. Significant work remains in reducing the morbidity and mortality associated with traumatic hemorrhagic in areas of primary prevention, early recognition and accurate diagnosis, resuscitation therapy with hemostasis, and determination of resuscitation endpoints.

## Supplementary Information

**Additional file 1. Video 1**. Subxiphoid view of the heart. Large pericardial effusion causing tamponade. L, liver; LV; left ventricle; PE, pericardial effusion; RV, right ventricle; T, thrombus.

**Additional file 2. Video 2**. Right thoracic view at the diaphragm with a right hemothorax. Thoracic spine visualized above the diaphragm. Normally, the thoracic spine is obscured by air within the lung. HT, hemothorax; L, liver; D, diaphragm; TS, thoracic spine; SS, spine sign.

**Additional file 3. Video 3**. Right upper quadrant viewof the abdomen. Anechoic hemoperitoneum in the hepatorenal space. HP, hemoperitoneum; L, liver; RL, right kidney.

**Additional file 4. Video 4**. Left upper quadrant viewof the abdomen. Anechoic hemoperitoneum in the splenorenal space. HP, hemoperitoneum; LK, left kidney; S, spleen.

**Additional file 5. Video 5.** Pelvic sagittal view. Anechoic hemoperitoneum cephalad and posterior to the bladder. HP, hemoperitoneum; B, bladder.

**Additional file 6. Video 6.** Pelvic transverse view. Anechoic hemoperitoneum posterior to the bladder. HP, hemoperitoneum; B, bladder.

**Additional file 7. Video 7**. TTE sagittal view of IVC long axis. IVC collapses > 50% with respiration provide insight into the fluid status of an adult trauma patient. IVC, inferior vena cava, HP, hepatic vein, RA, right atrium, L, liver.

**Additional file 8. Video 8**. TEE transgastric short axis view.diastole;systole. Severe left ventricular hypovolemia and papillary muscle kissing sign during systole. LV, left ventricle; RV, right ventricle.

**Additional file 9. Video 9**. Apical 4-chamber view. Intravascular volume status and function: Reduced fractional area change indicating both RV and LV dysfunction. LV, left ventricle; MV, mitral valve; RV, right ventricle.

**Additional file 10: Table S2**. Classification of Hemorrhagic Shock.

## Data Availability

Data sharing not applicable to this article as no datasets were generated or analyzed during the current study.
