# Supplementary figures and images for: Traumatic hemorrhage and chain of survival
Source: Scand J Trauma Resusc Emerg Med. 2023 May 24;31:25. doi: 10.1186/s13049-023-01088-8 (PMC10207757; doi:10.1186/s13049-023-01088-8)

## Slide 1
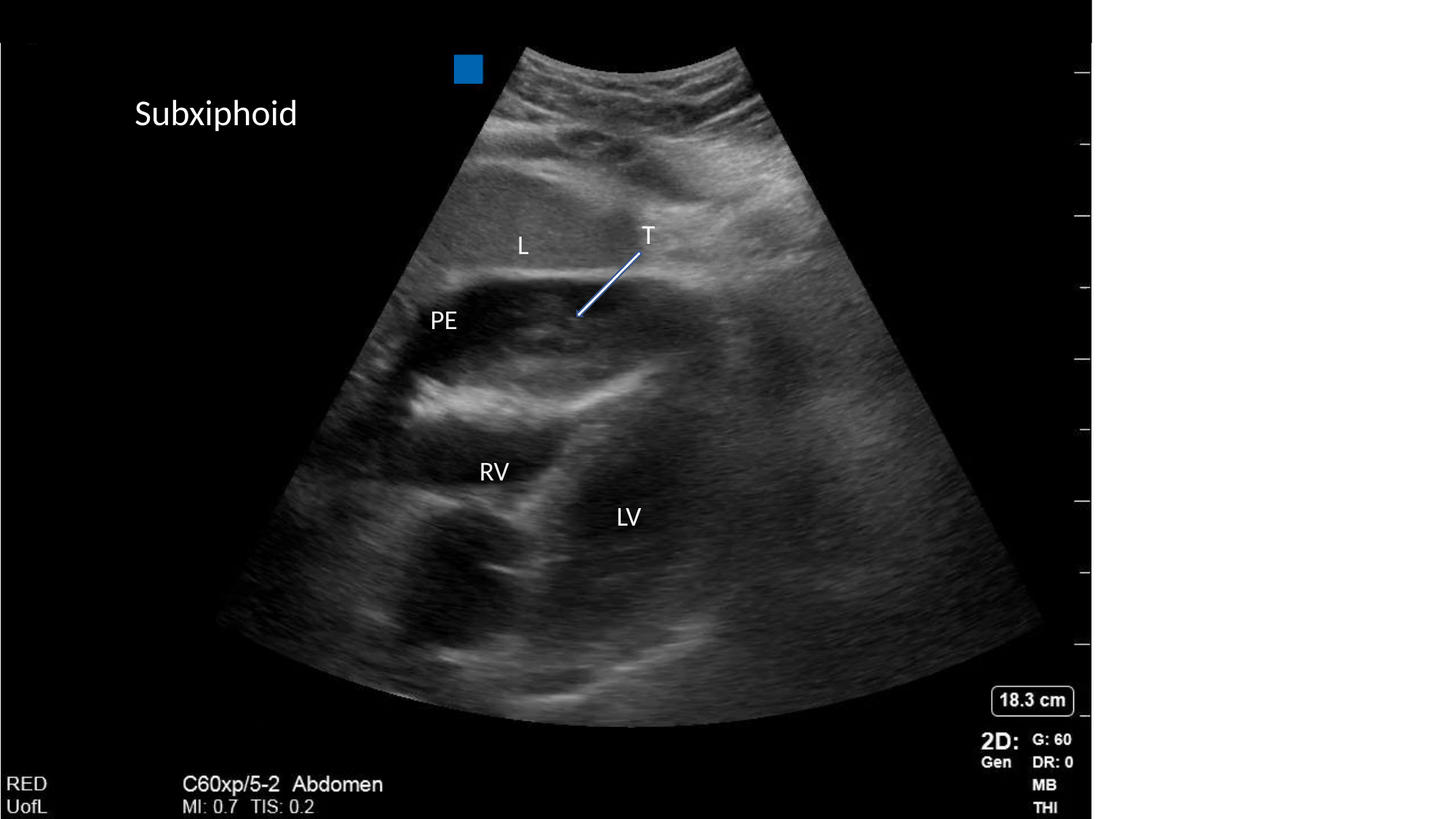

Subxiphoid
T
L
PE
RV
LV

Supplement: Supplementary file 1 — Additional file 1. Video 1. Subxiphoid view of the heart. Large pericardial effusion causing tamponade. L, liver; LV; left ventricle; PE, pericardial effusion; RV, right ventricle; T, thrombus. [file 13049_2023_1088_MOESM1_ESM.pptx]

## Slide 1
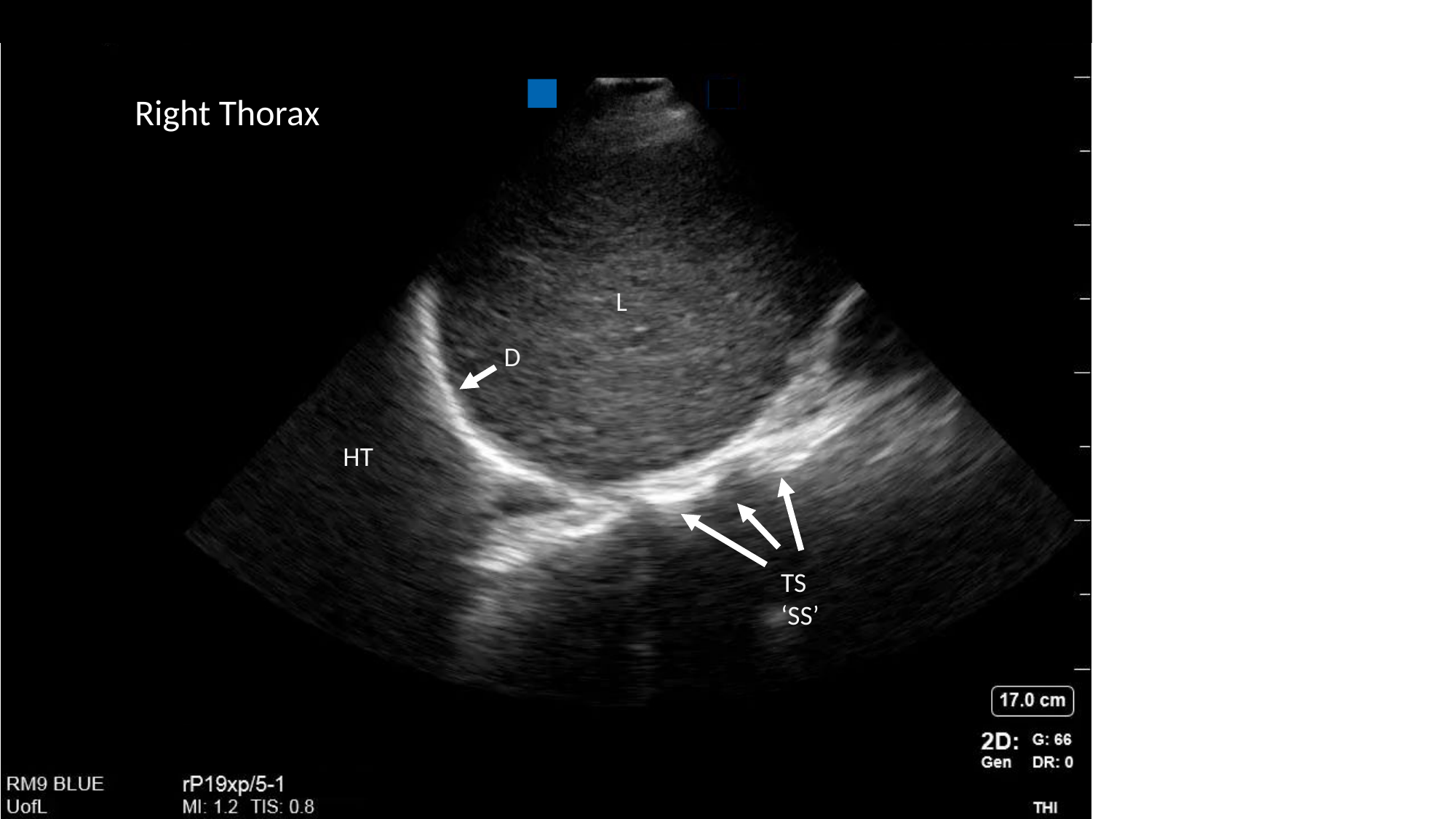

Right Thorax
L
D
HT
TS
‘SS’

Supplement: Supplementary file 2 — Additional file 2. Video 2. Right thoracic view at the diaphragm with a right hemothorax. Thoracic spine visualized above the diaphragm. Normally, the thoracic spine is obscured by air within the lung. HT, hemothorax; L, liver; D, diaphragm; TS, thoracic spine; SS, spine sign. [file 13049_2023_1088_MOESM2_ESM.pptx]

## Slide 1
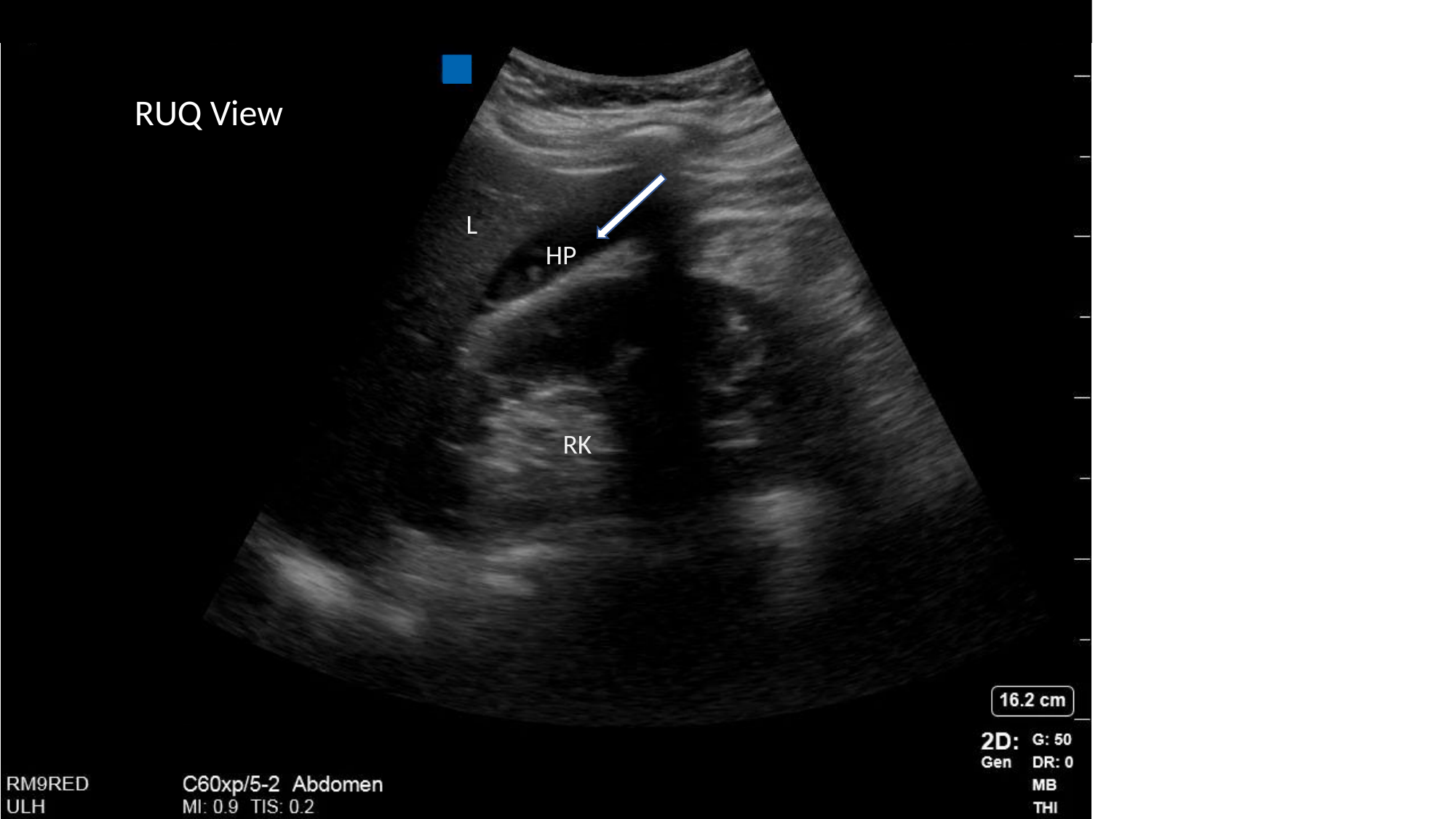

RUQ View
L
HP
RK

Supplement: Supplementary file 3 — Additional file 3. Video 3. Right upper quadrant viewof the abdomen. Anechoic hemoperitoneum in the hepatorenal space. HP, hemoperitoneum; L, liver; RL, right kidney. [file 13049_2023_1088_MOESM3_ESM.pptx]

## Slide 1
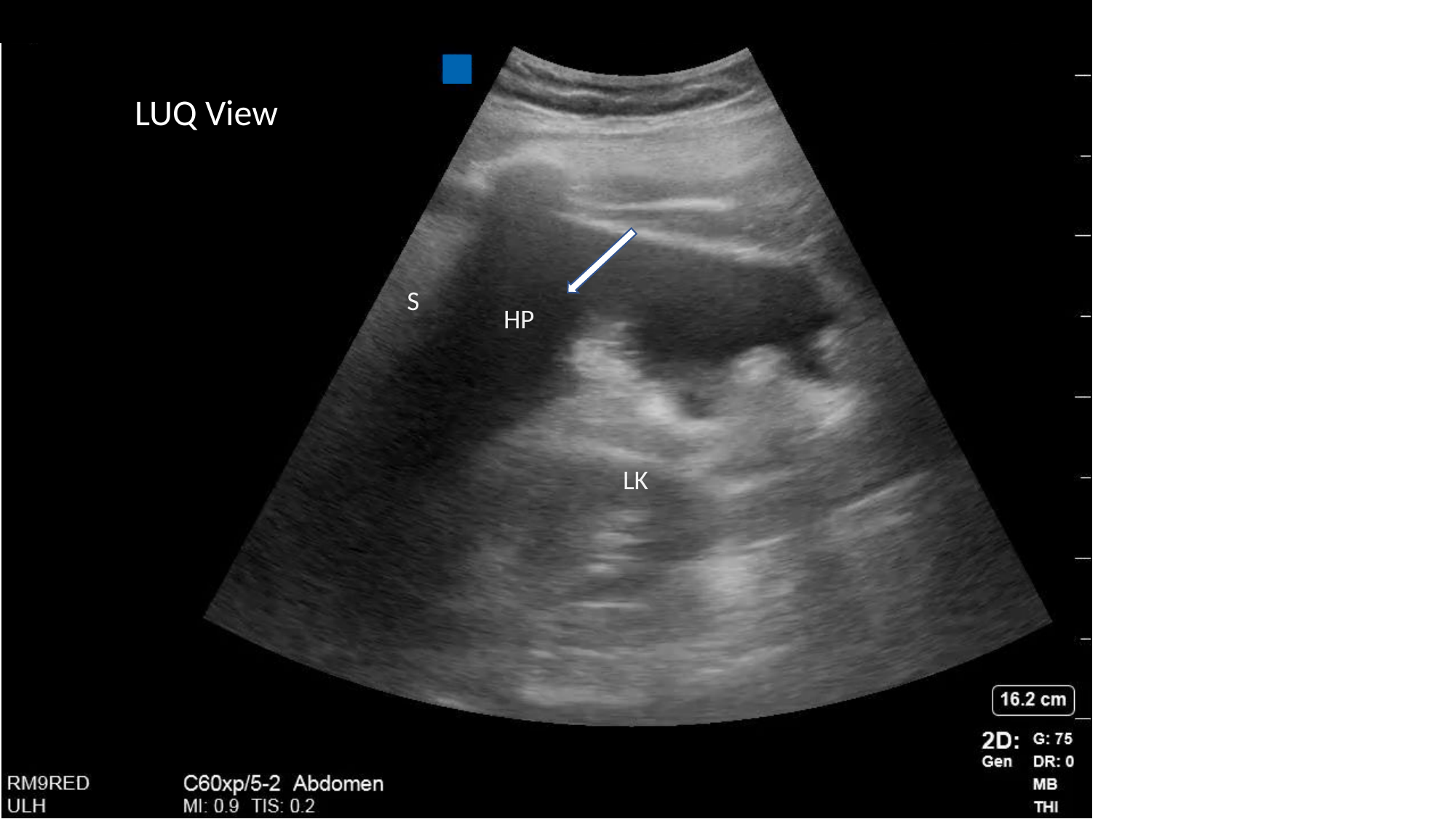

LUQ View
S
HP
LK

Supplement: Supplementary file 4 — Additional file 4. Video 4. Left upper quadrant viewof the abdomen. Anechoic hemoperitoneum in the splenorenal space. HP, hemoperitoneum; LK, left kidney; S, spleen. [file 13049_2023_1088_MOESM4_ESM.pptx]

## Slide 1
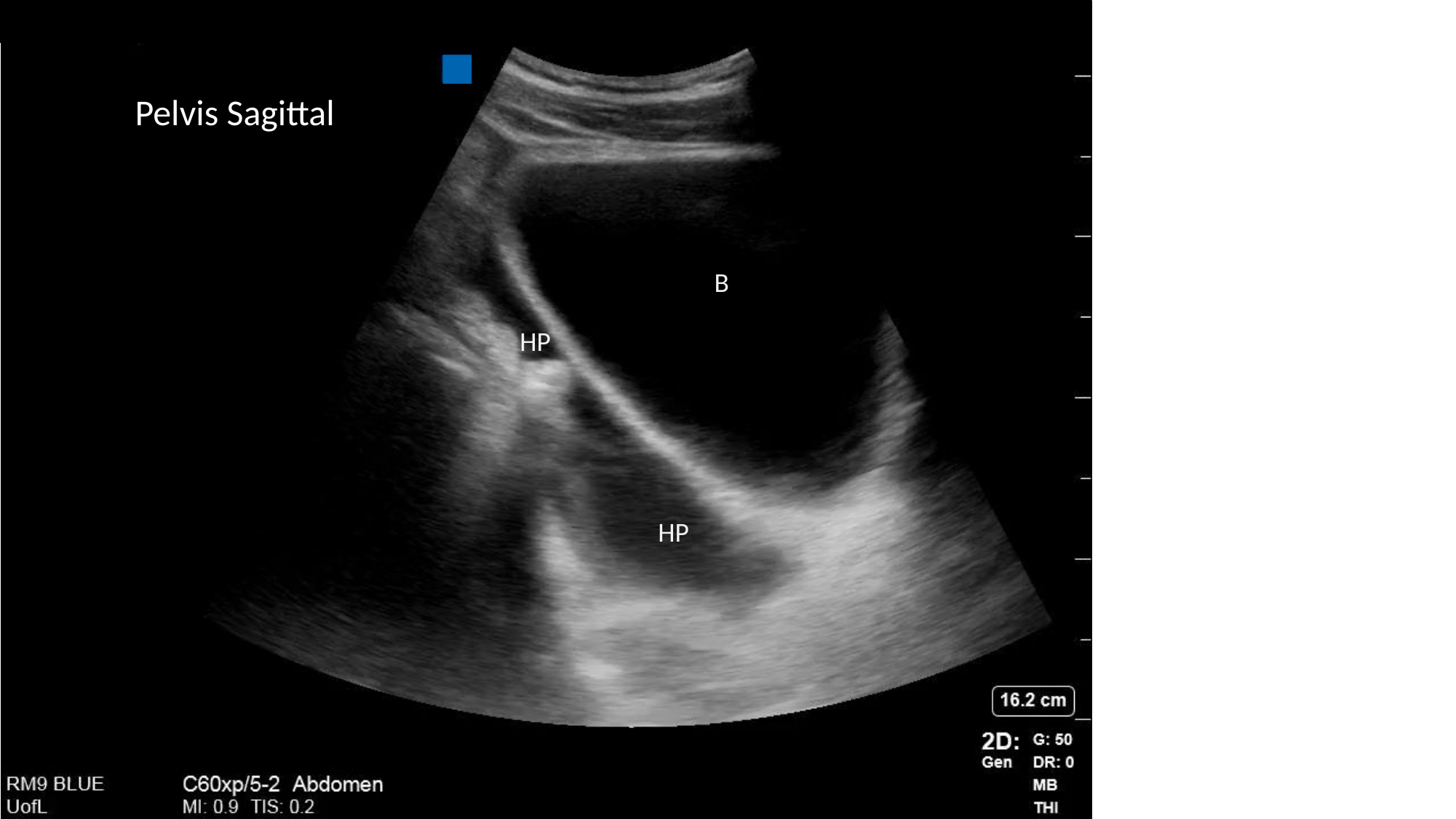

Pelvis Sagittal
B
HP
HP

Supplement: Supplementary file 5 — Additional file 5. Video 5. Pelvic sagittal view. Anechoic hemoperitoneum cephalad and posterior to the bladder. HP, hemoperitoneum; B, bladder. [file 13049_2023_1088_MOESM5_ESM.pptx]

## Slide 1
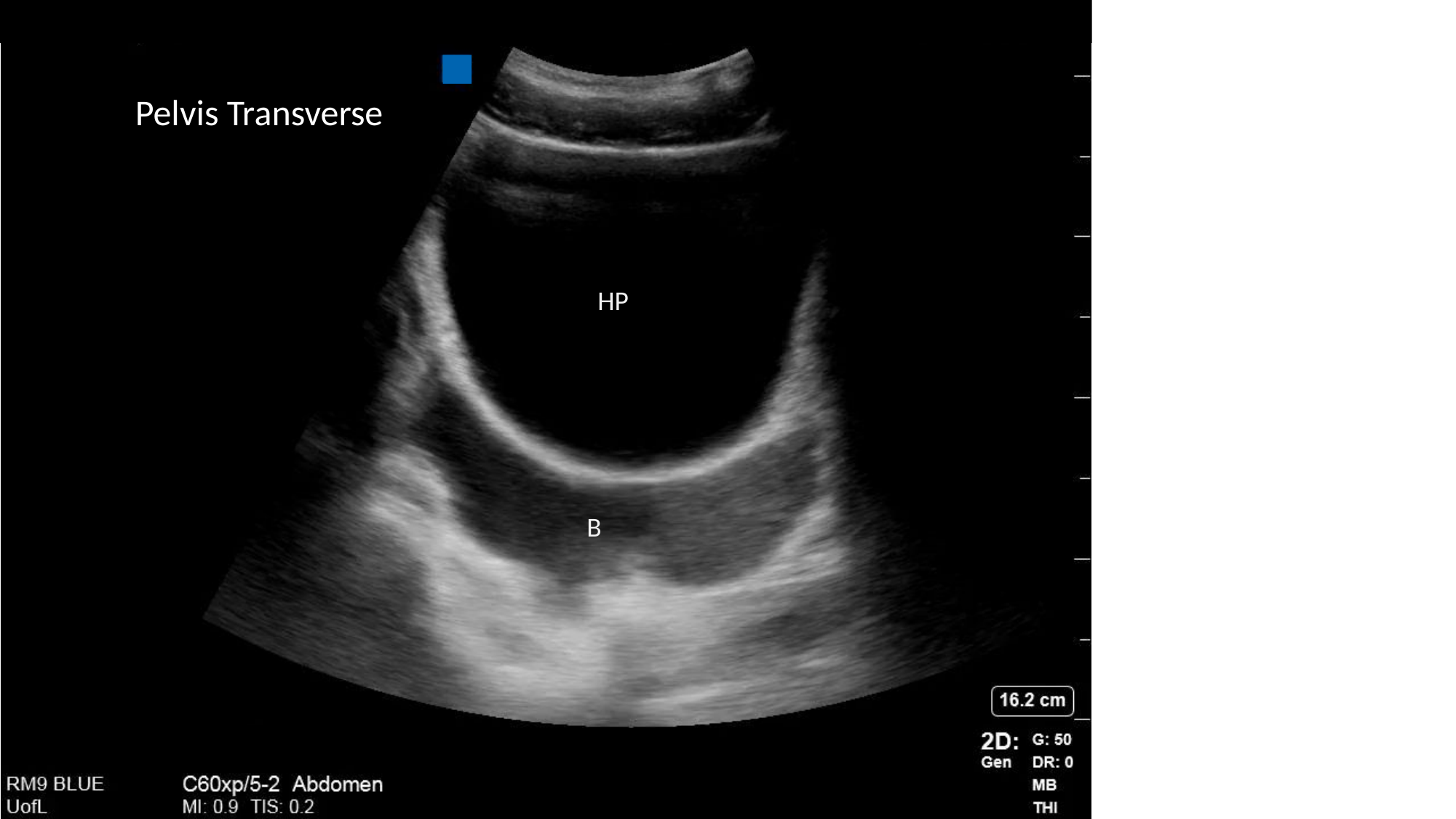

Pelvis Transverse
HP
B

Supplement: Supplementary file 6 — Additional file 6. Video 6. Pelvic transverse view. Anechoic hemoperitoneum posterior to the bladder. HP, hemoperitoneum; B, bladder. [file 13049_2023_1088_MOESM6_ESM.pptx]

## Slide 1
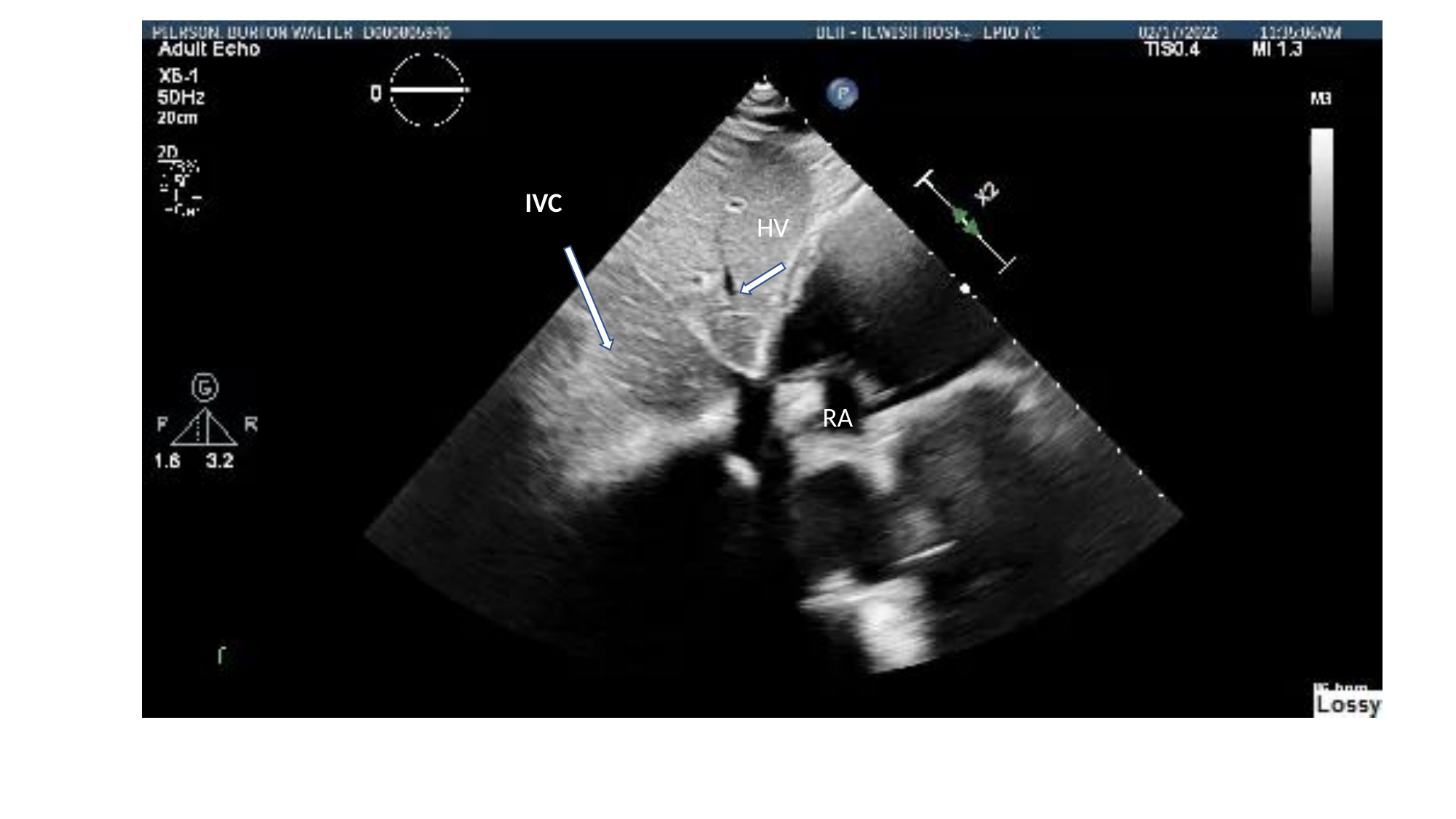

IVC
HV
RA

Supplement: Supplementary file 7 — Additional file 7. Video 7. TTE sagittal view of IVC long axis. IVC collapses > 50% with respiration provide insight into the fluid status of an adult trauma patient. IVC, inferior vena cava, HP, hepatic vein, RA, right atrium, L, liver. [file 13049_2023_1088_MOESM7_ESM.pptx]

## Slide 1
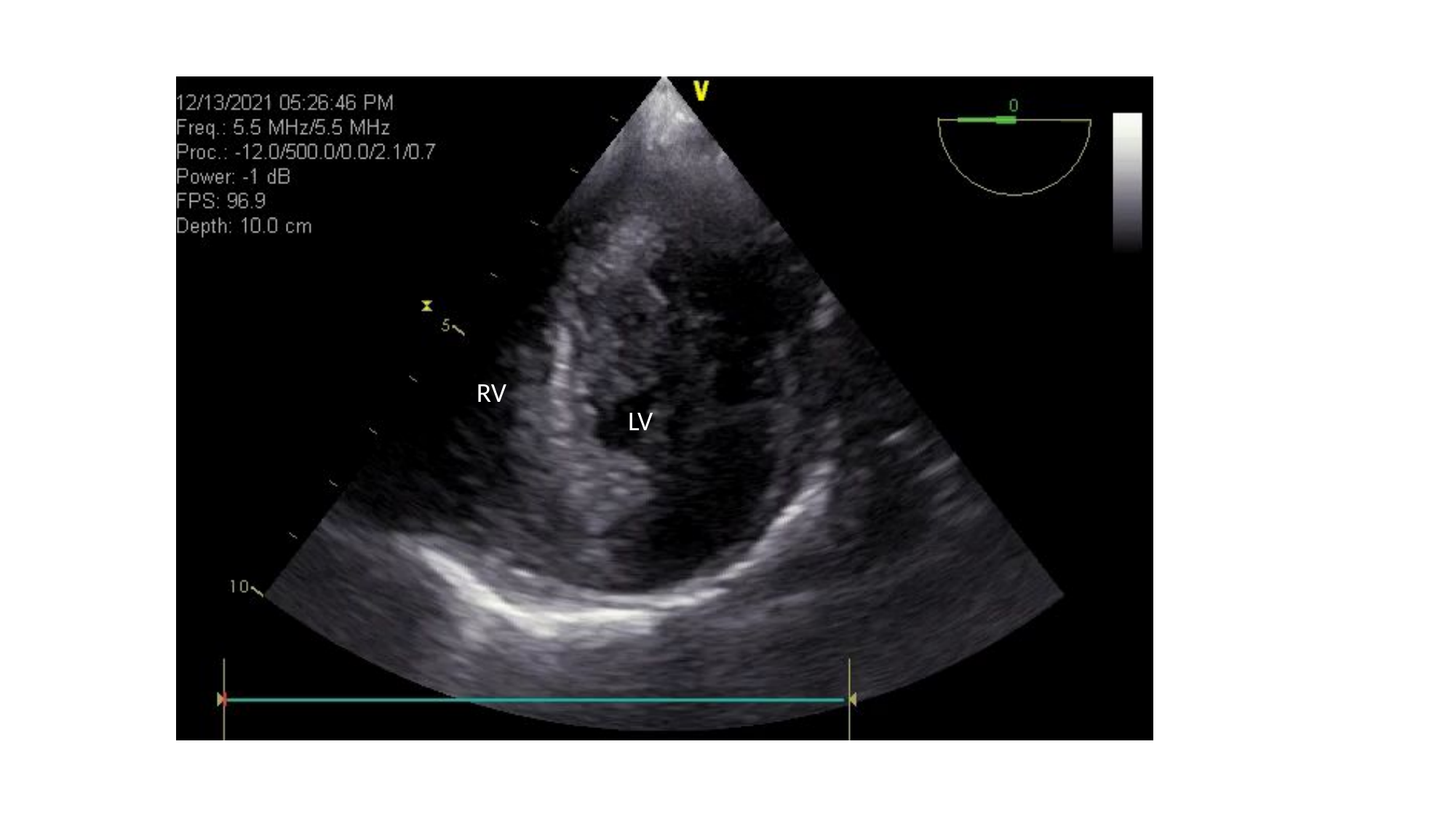

RV
LV

Supplement: Supplementary file 8 — Additional file 8. Video 8. TEE transgastric short axis view.diastole;systole. Severe left ventricular hypovolemia and papillary muscle kissing sign during systole. LV, left ventricle; RV, right ventricle. [file 13049_2023_1088_MOESM8_ESM.pptx]

## Slide 1
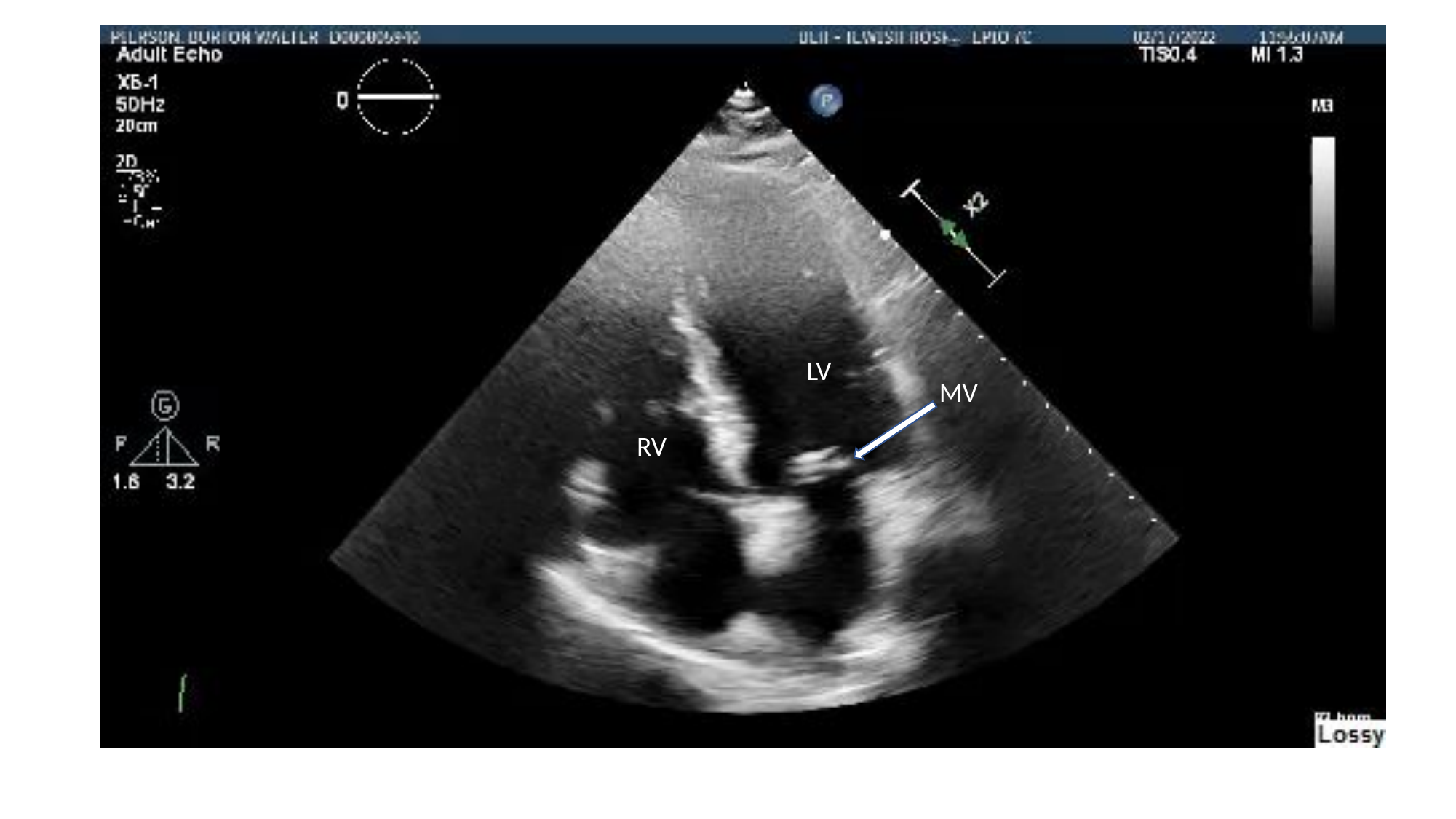

LV
MV
RV

Supplement: Supplementary file 9 — Additional file 9. Video 9. Apical 4-chamber view. Intravascular volume status and function: Reduced fractional area change indicating both RV and LV dysfunction. LV, left ventricle; MV, mitral valve; RV, right ventricle. [file 13049_2023_1088_MOESM9_ESM.pptx]
