# Supplementary material for: Traumatic hemorrhage and chain of survival
Source: Scand J Trauma Resusc Emerg Med. 2023 May 24;31:25. doi: 10.1186/s13049-023-01088-8 (PMC10207757; doi:10.1186/s13049-023-01088-8)
Supplement: Supplementary file 10 — Additional file 10: Table S2. Classification of Hemorrhagic Shock. [file 13049_2023_1088_MOESM10_ESM.docx]

Supplementary Material

**Table S1. Classification of Hemorrhagic Shock. ᶲ**

| **Shock Class** | **I** | **II** | **III** | **IV** |
| --- | --- | --- | --- | --- |
| Blood loss (mL) ‡ | Up to 750 ml | 750-1500 | 1500-2000 | >2000 |
| Blood loss (% volume) ‡ | Up to 15 | 15-30 | 30-40 | >40 |
| Pulse rate (per minute) | < 100 | 100-120 | 120-140 | >140 |
| Blood Pressure | Normal | Normal | Decreased | Decreased |
| Pulse Pressure (mm Hg) | Normal or increased | Decreased | Decreased | Decreased |
| Respiratory Rate (per min) | 14-20 | 20-30 | 30-40 | >35 |
| Mental Status | Slightly anxious | Mildly anxious | Anxious, confused | Confused, lethargic |

ᶲData are from the American College of Surgeons Committee on Trauma.

‡Blood-loss volume and percentage of total blood volume are for a male patient with a body weight of 70 kg
